# Serum and Tissue Level of TLR9 in EBV-Associated Oropharyngeal Cancer

**DOI:** 10.3390/cancers13163981

**Published:** 2021-08-06

**Authors:** Ewa Stępień, Małgorzata Strycharz-Dudziak, Maria Malm, Bartłomiej Drop, Małgorzata Polz-Dacewicz

**Affiliations:** 1 Department of Virology with SARS Laboratory, Medical University of Lublin, 20-059 Lublin, Poland; ewa.stepien@umlub.pl (E.S.); m.polz@umlub.pl (M.P.-D.); 2 Chair and Department of Conservative Dentistry with Endodontics, Medical University of Lublin, 20-059 Lublin, Poland; 3 Department of Information Technology and Medical Statistics, Medical University of Lublin, 20-059 Lublin, Poland; maria.malm@umlub.pl (M.M.); bartlomiej.drop@umlub.pl (B.D.)

**Keywords:** oropharyngeal cancer, EBV, TLR9, cytokines

## Abstract

**Simple Summary:**

The Epstein–Barr virus (EBV) is associated with the development and progression of various epithelial malignancies including cancer in the head and neck region. Toll-like receptors (TLRs) are molecules distinguishing self and non-self antigens. They are required for congenital immune response to infections with viruses such as EBV because, during viral infection, the congenital immunity is the first line of human defense preventing the replication of the virus. Moreover, TLR response may influence the transformation to malignancy. The aim of our study was to assess TLR9 level in patients with diagnosed oropharyngeal cancer with or without EBV infection. We wanted to know whether infection with EBV influences TLR9 level and maybe changes the immune response which may lead to malignant transformation. The results obtained in our research may improve understanding of the role viral infections play in head and neck cancers and influence future diagnosis, prevention and treatment strategies in these malignancies.

**Abstract:**

The Epstein–Barr virus (EBV) is associated with the development of various epithelial malignancies including cancer in the head and neck region. Several studies have shown that Toll-like receptors (TLRs) are required for an innate immune response to infection with human DNA viruses, e.g., EBV. During viral infections, TLR response may influence the transformation to malignancy. The aim of the study was to assess TLR9 serum and tissue level in EBV(+) and EBV(−) oropharyngeal cancer patients. The study involved 78 patients: 42 EBV(+) and 36 EBV(−). EBV DNA was detected in fresh frozen tumor tissue. TLR9 level was measured in homogenate of tumor tissue and in serum. Moreover, in serum samples IL-10, VEGF, TGFβ, TNFα and antibodies against EBV were detected using ELISA test. TLR9 level was significantly lower in EBV(+) patients, both in tissue and serum, while EBVCA, EBNA and VEGF level was statistically higher in EBV(+) patients. An increase in EBVCA and EBNA antibodies titer was correlated with a TLR9 level decrease. TLR9 level was higher in poorly-differentiated tumors (G3), in tumor of larger dimensions (T3-T4) and with lymph nodes involvement (N3-N4) but without statistical significance. High levels of anti-EA antibodies in the majority of EBV(+) patients may point to the reactivation of EBV infection.

## 1. Introduction

More than 90% of cancers arising in the head and neck region develop from the epithelium in the oral cavity, pharynx and larynx. These malignancies are a serious worldwide health problem as globally per year over 550,000 patients are diagnosed with head and neck squamous cell carcinoma (HNSCC) and more than 380,000 deaths are reported due to this disease [1].

The Epstein–Barr virus (EBV) is a widespread gammaherpesvirus infecting the vast majority of the world human population. It is also the first known human virus with carcinogenic potential and infection with EBV has been associated with the development and progression of various cancers derived from B cells, e.g., Burkitt’s lymphoma, Hodgkin’s lymphoma, but also with epithelial malignancies such as gastric cancer and nasopharyngeal cancer (NPC) [2,3,4,5,6]. EBV DNA was also detected in oropharyngeal cancer [7,8,9]. Subsequent to primary infection, EBV establishes latency–persistent infection in affected cells–and can reactivate periodically by entering the lytic cycle with viral transmission in the head and neck epithelium, thus affecting the pathogenesis of tumors associated with EBV. The switch from latent to lytic phase is a crucial event in EBV infection, however, the mechanism is still not elucidated [6,10,11].

Evasion of the immune system and chronic infection are essential steps for diseases associated with EBV. Recent studies indicate that the host’s immunological responses to viruses play an important role in cancer development, and that tumor progression could partly be due to a failure in the innate immune response [12,13]. During viral infections, congenital immunity is the first line of human defense preventing the replication of the virus. Various microorganisms may be recognized by pattern recognition-receptors (PRR) which can distinguish specific molecules or structures unique to this pathogen known as pathogen-associated molecular patterns (PAMPs). One of PRR representatives are Toll-like receptors (TLR), the most common family of congenital sensors with ability to recognize a wide range of PAMPs and host associated molecules causing damage, which are crucial in recognizing infection and initiating the immunological response. TLR activity following viral stimulation results in a rapid production of inflammatory cytokines and antiviral mediators. Among ten different TLRs characterized in humans, some are expressed on the cell surface, while other (e.g., TLR9) are almost exclusively expressed in intracellular compartments. The human TLR9, is a DNA receptor recognizing unmethylated nucleic acid containing Cytosine-phosphate-Guanine (CpG) motifs present in bacteria and viruses [14,15,16].

Various researches revealed that TLR9 are important factor mediating interaction between EBV and the host immune response [17,18]. Moreover, during viral infections, the role and response of TLR may influence transformation to malignancy. Therefore, the aim of the study was to assess the level of TLR9 in the serum and tissue of patients with EBV(+) and EBV(−) oropharyngeal squamous cell carcinoma (OPSCC). Additionally, the level of selected cytokines, such as IL-10, VEGF, TGFβ, TNFα and antibodies against EBV was also determined. Moreover, the relationship between TLR9 levels and TN classification and histological differentiation of the tumor (G) was analyzed as well as the correlation between serum level of TLR9 and examined cytokines.

## 2. Materials and Methods

### 2.1. Patients

The present study involved 78 patients with a diagnosed and histopathologically confirmed OPSCC, who were hospitalized at the Otolaryngology Division of the Masovian Specialist Hospital in Radom, Poland. The patients had not received radiotherapy or chemotherapy before. This group consisted of 42 EBV(+) and 36 EBV(−) patients. In terms of socio-demographic features, smoking and alcohol consumption these groups did not differ and therefore the features did not affect the values of the examined parameters. Clinical and epidemiological characteristics of the patients are presented in Table 1.

The research was approved by the Medical University of Lublin Ethics Committee and is in accordance with GCP (Good Clinical Practice) regulations (No. KE-0254/295/2019, 26 September 2019). Informed written consent was collected from all participants.

### 2.2. Clinical Specimens

The tissue samples were collected from all patients during surgery and frozen at −80 °C until analysis. Tumor, node, metastasis (TNM) classification was determined during primary diagnosis according to the criteria of the Union for International Cancer Control (UICC) [19]. Histological grading was performed according to World Health Organization criteria, which divide tumors into three types: well differentiated (G1), moderately differentiated (G2) and poorly differentiated (G3) [20].

### 2.3. Serum Collection

Venous blood samples collected from all patients were centrifuged at 1500 rpm for 15 min at room temperature and the serum was frozen at −80 °C until analysis.

### 2.4. Molecular Methods

#### 2.4.1. DNA Extraction from Fresh Frozen Tumor Tissue

Fragments of the fresh frozen tumor tissue (20 mg) from the patients with OPSCC were cut and homogenized in a manual homogenizer Omni TH (Omni International, Kennesewa, GA, USA). DNA was extracted using a protocol as described in the DNeasy Tissue Kit Handbook (QiagenGmBH, Hilden, Germany). Purified DNA was quantified by spectrophotometry (Epoch Microplate Spectrophotometer, BioTek Instruments Inc., Vinooski, VT, USA). The isolated genetic material was kept at −20 °C until the test was conducted. To verify the quality of the obtained DNA (presence of inhibitors of polymerase chain reaction), a β-globin assay was performed.

#### 2.4.2. Detection of EBV DNA

EBV DNA detection and the amplification of the Epstein–Barr nuclear antigen 2 (EBNA-2) gene (the nested PCR) were performed as previously described [21]. Briefly, the nested PCR was carried out for amplification of Epstein–Barr nuclear antigen 2 (EBNA-2). The sequence of primers used for PCR was as follows: outer pair 5′-TTT CAC CAA TAC ATG ACC C-3′, 5′-TGG CAA AGT GCT GAG AGC AA-3′ and inner pair 5′-CAA TAC ATG AAC CRG AGT CC-3′, 5′-AAG TGC TGA GAG CAA GGC MC-3′.

All PCR reactions were carried out in the final volume of 25 μL using HotStartTaq DNA Polymerase (Qiagen, Hilden, Germany). Concentrations of PCR reaction components were prepared as follows: 2.0 mM MgCl_2_, 0.2 mM dNTPs, 0.5 μM of each forward and reverse primers and 0.5 U of HotStartTaq polymerase. During each run the samples were tested together with one negative (nuclease-free water) and positive control (EBV-positive cell line, Namalwa, ATCC-CRL-1432).

#### 2.4.3. Genotyping of LMP1

PCR screening for the EBV LMP1 subtype based on exon 3, defined as wild-type (wtLMP1) or del-LMP1, was done using specific primers: forward 5′-AGC GAC TCT GCT GGA AAT GAT-3′; reverse 5′-TGA TTA GCT AAG GCA TTC CCA-3′. Concentrations of PCR reaction components were prepared as follows: 2.0 mM MgCl_2_, 0.2 mM dNTPs, 0.5 μM of each forward and reverse primers and 0.5 U Hot Start DNA polymerase and 5 μL of extracted DNA. The reaction mixture (25 μL) was incubated at 95 °C for 15 min., followed by 45 cycles at 94 °C for 1 min., 57 °C for 1 min., 72 °C for 1 min., a final extension at 72 °C for 10 min. PCR products were analyzed by gel electrophoresis in a 3% agarose gel and visualized under UV light.

### 2.5. Serological Methods

#### 2.5.1. Identification of EBV Antibodies

In the collected serum samples, serological tests for antibodies against EBV were conducted by means of the immunoenzymatic ELISA method, using the commercially available ELISA tests as previously described [21].

Designed antibodies included: anti-VCA IgM (Nova-Lisa Epstein–Barr Virus VCA IgM/Nova Tec Immunodiagnostica GmbH/Germany/catalog number: EBVM0150), anti-VCA IgG (NovaLisa Epstein–Barr Virus VCA IgG/Nova Tec Immunodiagnostica GmbH/Germany/catalog number: EBVG0150) and anti-EBNA IgG (NovaLisa Epstein–Barr Virus EBNA IgG/Nova Tec Immunodiagnostica GmbH/Germany/catalog number: EBVG0580), antibodies anti-EA IgG (ELISA-VIDITEST anti-EA (D) EBVIgG/Vidia/Czech Republic/catalog number: ODZ-006).

All tests were performed according to the manufacturer’s instructions. The NovaTec Epstein–Barr Virus (EBV) IgG-ELISA is intended for the qualitative determination of IgG class antibodies against Epstein–Barr virus. Samples were considered positive if the absorbance value was higher than 10% over the cut-off. The level of antibodies was expressed as NovaTec-Units = NTU. ELISA-VIDITEST anti-EA is a semiquantitative test. Samples with absorbances higher than 110% of the cut-off value were considered positive.

#### 2.5.2. Measuring of Cytokines Level

The level of IL-10, TNF-α, TGF-β and VEGF were established in sera of patients by ELISA (enzyme-linked immunosorbent assay) using commercially available kits Diaclone SAS, France. The cytokine level of each serum sample was tested three times and the results are the mean values of these. The level of tested cytokines was expressed in pg/mL or ng/mL. The minimum detectable dose of IL-10 was less than 0.98 pg/mL (IL-10 HS ELISA kit) cat. no. 850.880.096; TNF-α–less than 8 pg/mL cat. no 950.090.096; TGF-β–8.6 pg/mL cat. No. 650.010.096 and VEGF–7.9 pg/mL cat. No. 650.080.096.

### 2.6. TLR9 Assay

Serum and tissue level of TLR9 was marked with a Cloud-Clone Corp. (Houston, TX, USA) (HEA709Hu) kit according to the manufacturer’s instruction. The kit is a sandwich enzyme immunoassay for in vitro quantitative measurement of TLR9 in human serum, plasma, tissue homogenates, cell lysates, cell culture supernates and other biological fluids. The concentration of high sensitive Toll-like receptor 9 (TLR9) in the samples was then determined by comparing the O.D. of the samples to the standard curve. Absorbance was measured in a spectrophotometer Epoch (Biotek Instruments, Winooski, VT, USA). The results were analyzed with the use of a software for the reader Gen 5 (Biotek Instruments, USA). The serum level of TLR9 is presented in pg/mL. The minimum detectable dose of this kit is typically less than 12.1 pg/mL.

TLRs concentrations in tissue homogenates were adjusted to total protein level and are expressed as pg/g of protein. To determine TLRs protein concentration, tissue samples were rinsed with 0.9% NaCl and stored at −80 °C until the analysis. Tissue homogenates (10% *w*/*v*) were prepared in 0.1 mol. l–1Tris-HCl buffer, pH = 7.4 using a laboratory MPW-120 homogenizer and supernatants were obtained by centrifugation at 5000× *g* for 30 min.

Protein was measured using the Bradford method (1976). The assays were performed with the use of spectrophotometer SPECORD M40 (Carl Zeiss, Jena, Germany).

### 2.7. Statistical Analysis

Descriptive statistics were used to characterize patient baseline characteristics. Pearson’s chi-square test was used to investigate the relationship between clinical and demographic parameters. The Mann–Whitney U-test was used to compare TLR9, antibodies and cytokine level in EBV(+) and in EBV(−) patients as well as the level of LMP1, TN classification and histological differentiation (G). The correlation between TLR9 level and antibodies and examined cytokines was assessed with a Spearman correlation rank test. Statistical significance was defined as *p* < 0.05.

## 3. Results

Clinical and epidemiological characteristics of the patients are presented in Table 1. No statistically significant differences were present between the examined groups of patients. Men predominated both in the EBV(+) and in the EBV(−) group (92.86% and 86.11%, respectively). The majority of patients, both EBV(+) and EBV(−), were older than 50 years of age, came from urban area and smoked cigarettes: 78.57% in the EBV(+) and 80.56% in the EBV(−) group. Wild-type-LMP-1 was significantly more common in patients with OPSCC (81%).

TLR9 level was significantly lower in EBV(+) than in EBV(−) patients, both in serum and in tissue, however, the tissue level of TLR9 was twice as high as the serum level: 346.68 pg/mL and 165.80 pg/mL, respectively (*p* < 0.0001) (Table 2).

In EBV(+) patients the levels of EBVCA, EBNA as well as VEGF were statistically higher than in the EBV(−) group. IL-10 and TNFα levels were higher and TGFβ was lower in EBV(+) group, but the differences were not statistically significant (Table 2). The majority of EBV positive patients (78.6%) had high levels of anti-EA antibodies. No IgM antibodies were found in any examined patient.

**Figure 1:**
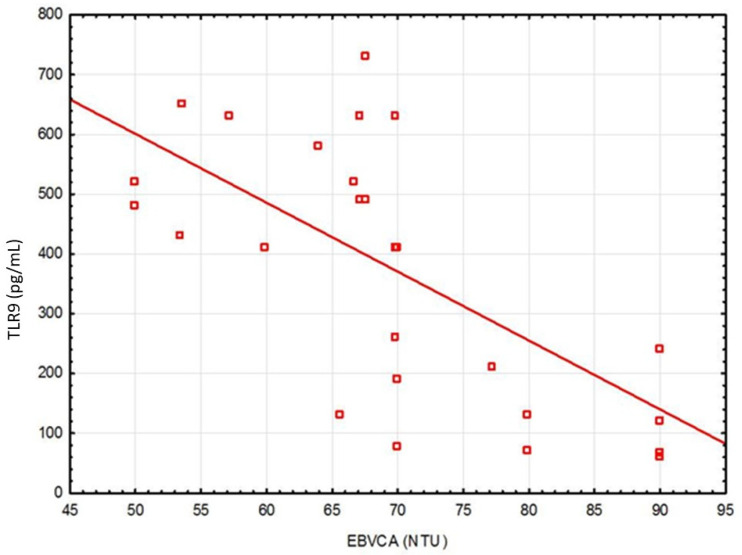
Correlation between serum level of TLR9 and EBVCA. Spearman correlation rank test (EBVCA&TLR9, r = −0.631462; *p* = 0.0001).

**Figure 2:**
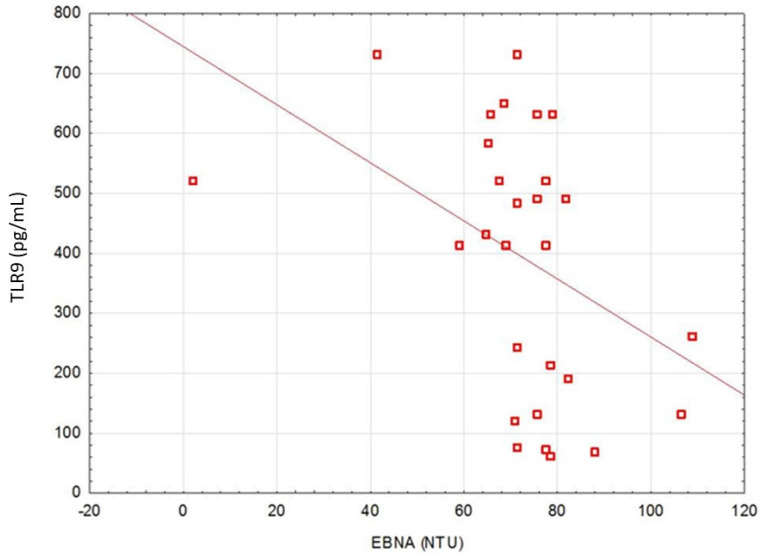
Correlation between serum level of TLR9 and EBNA. Spearman correlation rank test (EBNA&TLR9, r = −0.4132; *p* = 0.02323).

A correlation between TLR9 level and anti-EBVCA and anti-EBNA antibodies was stated (Figure 1 and Figure 2). An increase in the antibodies’ titer was accompanied by a decrease in TLR9 level.

TLR9 level was significantly higher in patients with del-LMP-1 than in patients infected with wild-type EBV, both in serum and in tissue (Table 3).

Analysis of TLR9 level and grading, as well as TN stage, revealed that TLR9 level was higher in poorly differentiated tumors (G3), in extensive tumor dimensions (T3–T4) and in lesions with lymph nodes involvement (N3–N4) but these differences were not statistically significant (Table 4).

**Figure 3:**
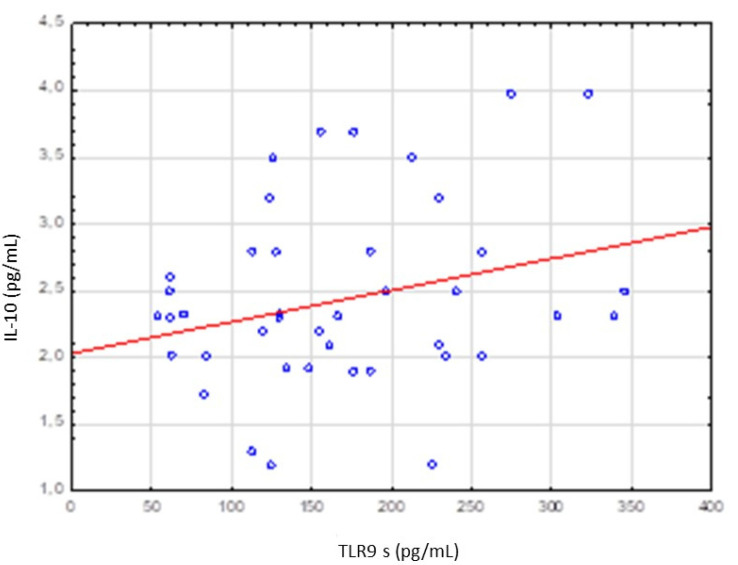
Correlation between serum levels of TLR9 and IL-10 level. Spearman rank test R = 0.226094 *p* = 0.149937.

**Figure 4:**
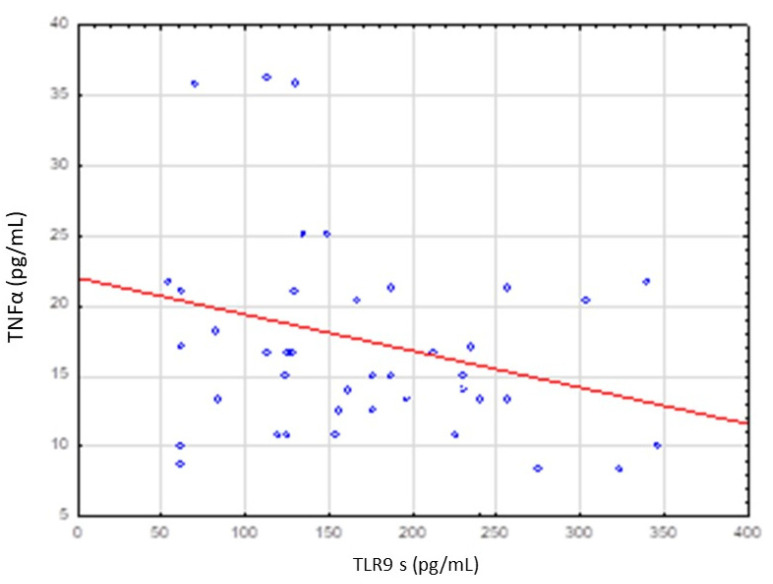
Correlation between serum level of TLR9 and TNFα level. Spearman rank test R = −0.235122; *p* = 0.133904.

No significant correlation was stated between TLR9 level and IL-10, TNFα, VEGF and TGFβ levels (Table 5). However, increase in TLR9 level was accompanied by increase in IL-10 level and decrease in TNFα level (Figure 3 and Figure 4).

## 4. Discussion

TLRs play a key role in the enhanced immune response against viruses [22,23]. Various studies have shown that TLR9 is required for the innate immune response to infections with human DNA viruses, including HSV-2 and EBV [24,25,26]. The activity of TLR9 is aimed at inducing the expression of pro-inflammatory cytokines against pathogens and as a result these receptors are inhibited by EBV so that it can continue to infect the human cells. Therefore, the analysis of TLR9-mediated antiviral cytokine secretion should be considered very important and, additionally, thorough understanding of the host’s innate immune response to EBV infection could explain how EBV can survive in order to affect cellular transformation [11,27]. There are some reports about TLR9’s role in immunity against EBV [25,28]. However, the literature investigating the relationships between TLR9 level, EBV infection and cytokine production in cancer is scarce.

Our study revealed that TLR9 level was significantly lower in EBV(+) than in EBV(−) patients, both in serum and in tissue. Several researchers suggest that EBV induces appropriate mechanism to suppress the response of major cells of the immune system [29,30,31]. According to Fathallah et al. [29], EBV may change the regulation and expression of TLR receptors, which are crucial effector molecules of the innate immune response. In their studies, infection of human primary B lymphocytes with EBV led to inhibition of TLR9 function, reducing the levels of mRNA and TLR9 protein, thus demonstrating that the virus decreases TLR9 level. Moreover, the authors stated that TLR9 stimulation on primary B cells uninfected with EBV led to the production of IL-6, TNF-α and IgG, which were inhibited in EBV-infected cells. Subsequently, the researchers noted that after EBV infection of the lymphoblastoid cell line, the level of TLR9 mRNA decreased for up to 120 h as compared to primary B lymphocytes not infected with EBV. This relationship was observed in the initial stages after infection of primary B cells with EBV, as well as in the immortalized lymphoblastoid cell line infected with EBV. It may point to a difference between viral escape to avoid recognition by TLR9 and EBV coexistence with TLR9 in the early stages of infection [29].

Other researchers demonstrated that EBV uses TLR9 to infect B lymphocytes by inducing their proliferation in the initial phase of infection, while in the later phase it lowers TLR9 expression to escape an immune response [32]. Similar studies on TLR9 level were conducted by Martin et al. [33] who observed that both UV-inactivated EBV and active EBV decreased TLR9 expression in B-cells. The ability to lower TLR9 expression appears to be a common feature among DNA cancer viruses: Hasan et al. [34] reported that the main oncoproteins of HPV16, E6 and E7, reduce the level of TLR9 in primary keratinocytes and B lymphocytes to suppress TLR9 in B cells. Interestingly, the low-risk HPV types that are normally associated with benign cervical lesions are unable to alter TLR9 expression, suggesting that the reduction in TLR9 level may only be associated with oncogenic viruses.

Younesi et al. [31] observed that latent EBV infection can significantly inhibit TLR9 induction. However, the authors state that viral infection was not associated with TLR9 expression and the mechanism is still unclear. Gent et al. [30] reported that BGLF5 protein during the lytic phase of EBV infection inhibits TLR9 regulation through RNA degradation in human B lymphocytes.

One research investigated also whether the inhibition of TLR9 expression by EBV is mediated by the main EBV oncoprotein LMP-1 (latent membrane protein 1). It was found that LMP-1 is a potent inhibitor of TLR9 transcription. The authors also analyzed the relationship between the LMP-1 type and the level of TLR9 in serum and tissue [29]. The study seems to be similar to our own research as our results revealed a relationship between LMP-1 type and the level of TLR9 in serum and tissue. In the wt-LMP-1 (wild type LMP-1) group, TLR9 level was significantly lower both in serum (154.56 pg/mL) and in tissue (323.72 pg/mL) compared to the del-LMP-1 (LMP-1 with deletion) group (serum-213.60 pg/L, tissue-444.25 pg/mL). It may be caused by the fact that the presence of LMP-1 deletion may eliminate EBV’s ability to induce TLR9 lowering. It was shown that the level of TLR9 in the tissue was significantly higher than in the serum, both in the group infected with wt-LMP-1 and with del-LMP-1. Our results indicated that, in patients with oropharyngeal carcinoma, wt-LMP-1 was more common (81%) than the type with a deletion (19%). However, the results are limited by the small size of the study group, which makes it impossible to draw conclusions about the presence of a specific type of LMP1 associated with OPSCC.

Our study also analyzed the relationship between the level of anti-EA antibodies and the LMP-1 type as well as the serum and tissue levels of TLR9. In the patients with anti-EA antibodies, the serum and tissue levels of TLR9 were significantly lower. Higher levels of anti-EA antibodies in patients indicate EBV reactivation, and thus the production of LMP-1 oncoprotein, which leads to lowering TLR9 level both in serum and in tissue.

LMP-1 may reduce TLR9 induction, affecting TLR9 transcript stability or direct inhibition of TLR9 transcription. One research revealed that inhibition of TLR9 expression by EBV is mediated by the LMP-1, since EBV without LMP-1 is impaired in its ability to lower the TLR9 level. LMP-1 may contribute to immune suppression at an early stage of infection by blocking the secretion of IFN-α and increasing the transcription of the anti-inflammatory cytokine IL-10 [35]. However, elucidating the exact role of LMP-1 in EBV-related carcinogenesis is difficult as LMP-1 can activate multiple signaling pathways. It can be seen that the pathways related to B cell proliferation and LMP-1-induced NF-kB signaling pathway, play an important role in EBV latency and survival. It was also demonstrated that LMP-1 is able to activate the NF-kB pathway in primary and immortalized B lymphocytes and thus inhibit TLR9 transcription. In contrast, inhibition of the NF-kB pathway by various means in EBV infected cells may increase TLR9 expression. Moreover, it was reported that del-LMP-1 without well-characterized CTAR1 and CTAR2 domains, which are important for the activation of NF-kB pathways, revealed decreased efficacy in lowering TLR9 level. The relationships presented by the researchers showed the consequences of the activation of the NF-kB pathway through the mechanism used by LMP-1 to regulate TLR9 expression [18,29,36].

TLR signaling initiated by various pathogens such as EBV triggers a cascade of signaling events that stimulate the production of pro-inflammatory cytokines and chemokines. Among them, TNF-α stimulates the growth of neoplastic cells, affects the stromal cells and enhances both metastasis and angiogenesis. Yang et al. suggested that the ability to respond to TLR ligands properly may be impaired by single nucleotide polymorphism in TLR genes, and that dysregulation of TLR signaling may contribute to an imbalance between pro- and anti-inflammatory cytokines, resulting in altered susceptibility to tumor growth [37,38]. Various studies reported that expression of EBV gene products is involved in the latency and lytic cycles of this virus in EBV-related cancers, e.g., in NPC [6,7,10]. Some of EBV viral gene products can induce or influence the production of inflammatory cytokines such as IL-10, IL-6 and TGF-β [37]. Both inflammatory and neoplastic cells and interactions between them are constantly being investigated, they produce a diverse set of cytokines and chemokines that mediates all stages of inflammation and influence tumor development and progression [38,39,40,41]. Increased expression of cytokines is a common phenomenon in tumor cell lines derived from many types of cancer [42,43,44]. Several studies reveal that a significant proportion of malignant tumors arise from infection and chronic inflammation [12,45,46]. One of the head and neck cancers, NPC, has been associated with the overexpression of numerous cytokines in NPC biopsies [37]. Huang et al. [47] reported that cytokine synthesis may contribute to lymphocyte infiltration and/or tumor growth during NPC development. Some studies point to increased expression of IL-10 in epithelial NPC cells and a relationship between serum IL-10 level and undifferentiated and clinically late stage of NPC [48], while other reports have not found such a relationship [49,50].

Our study did not reveal a statistically significant relationship between TLR9 level and cytokines concentration. However, a kind of tendency in correlation between IL-10 and TLR9 levels, as well as between TNFα and TLR9 levels, was observed, therefore it would be beneficial to perform similar studies including larger group of patients. The majority of reports cited in our research analyzed TLR expression with PCR, while our study involved the assessment of TLR9 level with sandwich enzyme immunoassay for the quantitative measurement of TLR9, which seems to be a reliable, simple and quick method of TLR9 determination in various clinical human samples. Further research is needed to elucidate the role of TLR9 in neoplastic transformation and its possible role as a biomarker. Moreover, molecular research focused on genetic and epigenetic changes could lead to better diagnosing and personalized therapy approaches.

## 5. Conclusions

The present study revealed that in EBV(+) patients the level of TLR9 (in serum and tissue) was significantly lower, while EBVCA, EBNA as well as VEGF levels were statistically higher than in EBV(−) patients. An increase in the EBVCA and EBNA antibodies titers was accompanied by a decrease in TLR9 level.

TLR9 level was significantly lower in patients infected with wild-type EBV than in patients with del-LMP-1 EBV.

TLR9 level was higher in poorly differentiated tumors (G3), in extensive tumor dimensions (T3–T4) and in lesions with lymph nodes involvement (N3–N4) but these differences were not statistically significant. No significant relationship was stated between TLR9 level and IL-10, TNFα, VEGF and TGFβ levels. However, an increase in TLR9 level was accompanied by an increase in IL-10 level and a decrease in TNFα level.

## Figures and Tables

**Table 1:** Epidemiological and clinical characteristics of the patients.

		EBV				*p*
		Positive		Negative		
		*n*	%	*n*	%	
Sex	Female	3	7.14	5	13.89	0.2724
	Male	39	92.86	31	86.11	
Age	<50	6	14.29	3	8.33	0.7065
	50–69	19	45.24	18	50.0	
	70+	17	40.48	15	41.67	
Place of residence	urban	29	69.05	21	58.33	0.3254
	rural	13	30.95	15	41.67	
Smoking	yes	33	78.57	29	80.56	0.8287
	no	9	21.43	7	19.44	
Alcohol abuse	yes	18	42.86	16	44.44	0.8879
	no	24	57.14	20	55.56	
G	G1	8	19.05	12	33.33	0.3323
	G2	32	76.19	22	61.11	
	G3	2	4.76	2	5.56	
T	T1-T2	24	57.14	20	96	0.1094
	T3-T4	18	42.86	16	4	
N	N1-N2	30	71.43	28	77.77	0.8900
	N3-N4	12	28.57	8	22.23	
M	M0	30	100.0	25	100.0	-
LMP-1	wt-LMP-1	34	81.0	-	-	-
	Del-LMP-1	8	19.0	-	-	

**Table 2:** Level of TLR9, EBVCA, EBNA, TGFβ, IL-10, VEGF and TNFα in EBV(+) and EBV(−) OPSCC patients.

Parameter	EBV(+)		EBV(−)	*p*
	$\bar{X}$ ± SD		$\bar{X}$ ± SD	
TLR9 serum	165.80 ± 79.87		328.21 ± 108.43	0.0001 *
TLR9 tissue	346.68 ± 159.95		696.58 ± 235.74	0.0001 *
EBVCA	71.26 ± 4.55		62.38 ± 1.62	10^−6^ *
EBNA	85.50 ± 8.61		61.90 ± 4.13	10^−6^ *
TGFβ	13.35 ± 12.12		16.55 ± 16.45	0.7370
IL-10	2.43 ± 0.70		2.32 ± 0.59	0. 6848
VEGF	656.57 ± 295.35		485.56 ± 392.01	0.0111 *
TNFα	17.64 ± 7.50		16.51 ± 10.09	0.3090
EA	N	%	-	-
High	33	78.6	-	-
Low	9	21.4	-	-

* Statistically significant; Mann–Whitney U-test; TLR9–Toll-like receptor 9; EBVCA–Epstein–Barr virus capsid antigen; EBNA–Epstein–Barr virus nuclear antigen; TGFβ–transforming growth factor β; IL-10–interleukin 10; VEGF–vascular endothelial growth factor; TNFα–tumor necrosis factor α; EA–early antigen.

**Table 3:** Level of TLR9 in tissue and serum in wt-LMP1 and del-LMP1 groups of OPSCC cancer patients.

Parameter	EBV(+)	
	TLR9 in Tissue $\bar{X}$ ± SD	TLR9 in Serum $\bar{X}$ ± SD
wt-LMP-1	323.72 ± 158.88	154.56 ± 78.58
del-LMP-1	444.25 ± 131.94	213.60 ± 70.91
*p*	0.019374 *	0.031867 *

* Statistically significant; wt-LMP-1–wild-type LMP-1. del-LMP-1–LMP-1 with deletion.

**Table 4:** Tissue and serum level of TLR9 according to G (grading) and TN stage in patients with EBV(+) and EBV(−) oropharyngeal cancer.

Parameter	EBV(+)		EBV(−)	
	TLR9 in Tissue $\bar{X}$ ± SD	TLR9 in Serum $\bar{X}$ ± SD	TLR9 in Tissue $\bar{X}$ ± SD	TLR9 in Serum $\bar{X}$ ± SD
G1	365.38 ± 174.48	176.56 ± 93.75	721.54 ± 208.81	349.65 ± 86.11
G2–G3	424.0 ± 101.78	190.54 ± 54.76	787.74 ± 184.17	303.24 ± 104.12
P	0.3585	0.4043	0.7372	0.3983
T1–T2	345.8 9 ± 144.81	155.61 ± 71.96	676.52 ± 257.52	308.18 ± 112.52
T3–T4	390.63 ± 130.04	171.09 ± 70.97	675.55 ± 256.02	324.51 ± 115.82
P	0.8545	0.8692	0.9087	0.5806
N1–N2	342.30 ± 133.02	152.61 ± 73.08	721.01 ± 181.60	343.71 ± 92.88
N3–N4	398.85 ± 165.56	189.97 ± 80.97	557.28 ± 295.10	271.83 ± 136.85
*p*	0.4750	0.4735	0.2571	0.5413

Mann–Whitney U-Test.

**Table 5:** Correlation between TLR9 serum level and IL-10, TNFα, VEGF, TGFβ levels in EBV(+) patients.

Parameter	Spearman’s Rank Test; Statistically Significant *p* < 0.05			
	N	R Spearman	t (N-2)	*p*
TLR9 s & IL-10	42	0.226094	1.46795	0.149937
TLR9 s & TNFα	42	−0.235122	−1.52994	0.133904
TLR9 s & VEGF	42	−0.038944	−0.24649	0.806565
TLR9 s & TGF	42	−0.169080	−1.08498	0.284427

## Data Availability

The data presented in this study are available in the article.
